# Aggregation-Induced Intermolecular Charge Transfer Emission for Solution-Processable Bipolar Host Material via Adjusting the Length of Alkyl Chain

**DOI:** 10.3390/molecules27228099

**Published:** 2022-11-21

**Authors:** Wei Jiang, Guimin Zhao, Wenwen Tian, Yueming Sun

**Affiliations:** School of Chemistry and Chemical Engineering, Southeast University, Nanjing 211189, China

**Keywords:** aggregation-induced intermolecular charge transfer emission, length of alkyl chain, glass transition temperature, carrier transport and balance, host material

## Abstract

Molecules with donor–spacer–acceptor configuration have been developed rapidly given their peculiar properties. How to utilize intermolecular interactions and charge transfers for solution-processed organic light-emitting diodes (OLEDs) greatly relies on molecular design strategy. Herein, soluble luminophores with D-spacer-A motif were constructed via shortening the alkyl chain from nonane to propane, where the alkyl chain was utilized as a spatial linker between the donor and acceptor. The alkyl chain blocks the molecular conjugation and induces the existence of aggregation-induced intermolecular CT emission, as well as the improved solubility and morphology in a solid-state film. In addition, the length of the alkyl chain affects the glass transition temperature, carrier transport and balance properties. The mCP-3C-TRZ with nonane as the spacer shows better thermal stability and bipolar carrier transport ability, so the corresponding solution-processable phosphorescent organic light-emitting diodes exhibit superior external quantum efficiency of 9.8% when using mCP-3C-TRZ as a host material. This work offers a promising strategy to establish a bipolar host via utilizing intermolecular charge transfer process in an aggregated state.

## 1. Introduction

For nearly two decades, organic light-emitting diodes (OLEDs) have been the focus of academic research and commercial application because of significant advantages, such as low driving voltage, fast response, high resolution and flexible display [1,2,3,4]. In general, the high-performance OLEDs strongly depend on the rational molecular design strategy and complicated vacuum-deposited technological process, which impose restriction on their further development [5,6,7,8]. In contrast, the simple solution-processing method, including spin coating and inkjet printing, manifests some unique advantages, such as low cost, high utilization of used materials and more attraction for mass fabrication and production of OLEDs [9,10,11,12,13]. In theory, thermally activated delayed fluorescence (TADF) OLEDs and phosphorescent OLEDs could realize an internal quantum efficiency of 100%; however, the fluorescence-quenching phenomenon usually occurs in TADF emitters and phosphorescent emitters [14,15,16,17]. Hence, these emitters often have to be doped into the appropriate host material to increase excitons utilization and improve solid-state emission efficiency. Excellent host materials should meet the conditions of high triplet energy (E_T_), compatible frontier molecular orbital (FMO) energy levels, good thermal stability and bipolar and balanced charge carrier transport property [18,19,20,21,22,23]. Recently, scientists have made great efforts to develop host materials, including D-π-A-type hosts, which feature intramolecular charge transfer (CT), and exciplex-type hosts, which utilize intermolecular charge transfer [24,25,26]. Nevertheless, the host with a structure of D-π-A redshifts the emission spectrum and lowers the E_T_. The physically mixed exciplex-type host usually contain two components, donor molecules and acceptor molecules, but phase separation is inevitable in the process of solution-processed film formation. Therefore, how to realize efficient intermolecular CT transition and improved film-forming property is still a challenge when designing the host material suitable for the solution process.

**Figure 1:**
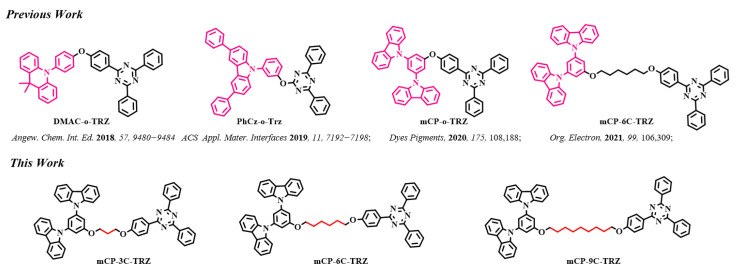
Molecular structure and design strategy of this work.

Lately, conjugation-forbidden linkage between D and A subunits is of increasing concern [27,28,29,30]. As shown in Figure 1, Wang et al. developed a TADF emitter (DMAC-o-TRZ) when oxygen atoms serve as a spacer between the DMAC donor unit and TRZ acceptor unit, strongly suppressing intramolecular CT transition and activating obvious intermolecular CT transition [28]. Duan et al. reported a TADF compound (PhCz-o-Trz) with the coexistence of both intra-CT and inter-CT in an amorphous aggregate, which could not only serve as an emitter but also as a sensitizing host, exhibiting high efficiency with alleviated efficiency roll-off [29]. Our group also designed two molecules with a D-σ-A motif and found that the mCP-o-TRZ molecule featured intramolecular CT and intermolecular CT but the TCz-o-TRZ molecule only owned intramolecular CT property [30]. Further, replacing the oxygen atom with an alkyl chain, three molecules showing only intermolecular CT process were prepared [31]. In the dilute solution, there only appears weak single-component emission, but stronger intermolecular charge transfer emission is induced due to closer D/A interaction in the aggregated state. The phenomenon is analogous to that of popular aggregation-induced emission (AIE) materials, coined by Tang’s group in 2001 [32,33,34,35,36], which show weak emission in an isolated state while emitting strongly in an aggregated state due to the restrictive intramolecular motion. Hence, it is theoretically feasible to design host materials with aggregation-induced intermolecular charge transfer emission. In addition, it is well-known that the alkyl chain could influence the carrier-charge mobility and π-π stacking interaction of organic semiconducting materials applied to an organic field-effect transistor or organic photovoltaics [37,38,39,40]. However, the effect of alkyl chain on the host material for solution-processed devices has not been revealed. For the solution method, the introduction of alkyl chain could improve the film-forming property, but it is not clear whether it influences the thermal stability and carrier transport characteristics or not?

In this work, three compounds were developed via adjusting the length of the alkyl chain from propane to nonane, where 1,3-di(9H-carbazol-9-yl) benzene (mCP) serves as donor group and 2,4,6-triphenyl-1,3,5-triazine (TRZ) acts as acceptor moiety, and alkyl chain as the spatial linker between mCP and TRZ units. The introduction of alkyl chain blocks the molecular conjugation, suppresses intramolecular CT process and induces the occurrence of aggregation-induced intermolecular CT transition, as well as the improved solubility and morphology. Although the alkyl chain could improve the film-forming property of materials, the longer the length of the alkyl chain, the lower the glass transition temperature. The length of alkyl chain hardly affects the excited state energy levels; all three molecules possess high E_T_, which is beneficial when acting as a host material. In addition, these three compounds show bipolar carrier transport characteristics due to the concurrence of electron-donating and electron-withdrawing groups in a single molecule. The shorter the alkyl chain, the more balanced the carrier transport. Finally, the solution-processed OLEDs employed mCP-3C-TRZ as a host for the phosphorescence guest (tris(2-methylphenylpyridine) iridium (III)) (Ir (mppy)_3_), realizing a maximum external quantum efficiency (EQE_max_) of 9.8%, which is superior to that of mCP-6C-TRZ and mCP-9C-TRZ.

## 2. Materials and Methods

### 2.1. Chemicals and Measurements

All compounds are commercially available from Chemical Company Ltd. and used in the reaction directly. All reactions were carried out under N_2_ atmosphere. The ^1^H NMR and ^13^C NMR spectra were acquired using a Bruker Dex-600/150 NMR instrument using CDCl_3_ as a solvent. Mass spectra (MS) were recorded by high-resolution mass spectrometer. Elemental analysis was determined by an Elementar Vario EL CHN (Elementar Analysensysteme GmbH, Langenselbold, Germany) elemental analyzer. UV-vis absorption spectra were recorded using a SHIMADZU UV-2600 spectrophotometer (SHIMADZU, Kyoto, Japan). Steady-state photoluminescence (PL) spectra were obtained with a HORIBA FLUOROMAX-4 spectrophotometer at room temperature with an excitation wavelength of 300 nm. The transient PL decay was obtained by EDIBURGH FLS-1000 instruments (EDIBURGH, Edinburgh, UK) at room temperature. The low-temperature phosphorescence (PH) spectra were recorded on F-7000 FL spectrophotometer after delayed 100 μs under liquid nitrogen. The solid PL quantum efficiency was measured using an integrating sphere under nitrogen at room temperature. Thermogravimetric analysis (TGA) and differential scanning calorimetry (DSC) curves were carried out with a Netzsch simultaneous thermal analyzer system (STA 409 PC, Burlington, MA, USA) from 20 °C to 800 °C and NETZSCH DSC 214 from 20 °C to 200 °C at a 10 °C/min heating rate under N_2_ atmosphere. Cyclic voltammetry measurements were performed using a CHI750C voltammetric analyzer (China). Electrochemical property was evaluated by cyclic voltammetry with three typical electrodes in dry CH_2_Cl_2_ solution (10^−3^ M) (oxidation process) with a rate of 100 mV/s. The CV system employed Bu_4_NPF_6_ as electrolyte. Platinum disk is used as the working electrode, platinum wire is regarded as the counter electrode and silver wire is used as the reference electrode. Ferrocenium/ferrocene (Fc/Fc^+^) was used as the external standard compound. All calculations were conducted using the Gaussian 09 program. The molecular orbitals were visualized using Gaussview 5.0 (Wallingford, CT, USA). The geometries were optimized at the B3LYP/6-31G(d) level. The transition energy and oscillator strength were calculated with TD-DFT method at the B3LYP/6-31G(d) level.

### 2.2. Synthesis

The synthesis of 9,9′-(5-(3-bromopropoxy)-1,3-phenylene)bis(9H-carbazole) (mCP-3C-Br) is as follows.

The OH-mCP (1.00 g, 2.36 mmol), 1,3-dibromopropane (1.00 g, 4.95 mmol) and cesium carbonate (2.31 g, 7.08 mmol) were placed in a 50 mL flask, then 20 mL DMF was poured into it and stirred under nitrogen for 90 min. After that, the mixture was poured into 60 mL water to precipitate the product. After separation and purification by chromatography column with the petroleum ether and dichloromethane, the clear mucus was obtained (1.03 g, 80%). Furthermore, ^1^H-NMR (600 MHz, CDCl_3_) δ = 8.15 (d, *J* = 7.7 Hz, 4H), 7.58 (d, *J* = 8.2 Hz, 4H), 7.45 (dd, *J* = 13.5, 5.2 Hz, 5H), 7.31 (t, *J* = 7.8 Hz, 4H), 7.24 (d, *J* = 8.6 Hz, 2H), 4.24 (t, *J* = 5.8 Hz, 2H), 3.65 (t, *J* = 6.4 Hz, 2H), 2.39 (d, *J* = 6.1 Hz, 2H).

The synthesis of 9,9′-(5-((9-bromononyl)oxy)-1,3-phenylene)bis(9H-carbazole) (mCP-9C-Br) is as follows.

The synthetic process of mCP-9C-Br was similar with that of mCP-3C-Br, with 1,3-dibromopropane replaced with 1,9-dibromononane. The clear mucus was obtained with a high yield of 75% (1.11 g). Furthermore, ^1^H-NMR (600 MHz, CDCl_3_) δ = 8.07 (d, *J* = 7.7 Hz, 4H), 7.50 (d, *J* = 8.2 Hz, 4H), 7.37 (t, *J* = 7.7 Hz, 4H), 7.32 (s, 1H), 7.23 (t, *J* = 7.4 Hz, 4H), 7.15 (d, *J* = 1.6 Hz, 2H), 3.99 (t, *J* = 6.5 Hz, 2H), 3.32 (t, *J* = 6.8 Hz, 2H), 1.84–1.72 (m, 4H), 1.48 (s, 2H), 1.41 (dd, *J* = 15.2, 7.7 Hz, 2H), 1.38–1.32 (m, 2H), 1.27 (dd, *J* = 14.0, 10.4 Hz, 4H).

The synthesis of 9,9′-(5-(3-(4-(4,6-diphenyl-1,3,5-triazin-2-yl)phenoxy)propoxy)-1,3-phenylene)bis(9H-carbazole) (mCP-3C-TRZ) is as follows.

The 9,9′-(5-(3-bromopropoxy)-1,3-phenylene)bis(9H-carbazole) (0.50 g, 0.92 mmol), cesium carbonate (0.90 g, 2.76 mmol), OH-TRZ (0.33 g, 1.00 mmol) and anhydrous DMF 10 mL were added to a round bottom flask (50 mL) under nitrogen atmosphere. The mixture was stirred at 25 °C for 6 h. After completion of the reaction, saturated salt water and ethyl acetate were added to the cooled mixture. The organic layer was separated, dried over MgSO_4_ and concentrated in vacuo. Later, the residue solid was purified by column chromatography to give the final product as a white powder (0.51 g, 70%). Furthermore ^1^H-NMR (600 MHz, CDCl_3_) δ = 8.73 (m, 6H), 8.14 (d, *J* = 7.7 Hz, 4H), 7.64–7.52 (m, 10H), 7.46–7.38 (m, 5H), 7.29 (m, 6H), 7.06 (d, *J* = 8.8 Hz, 2H), 4.34 (d, *J* = 14.6 Hz, 4H), 2.42 (t, *J* = 12.0 Hz, 2H). Furthermore, ^13^C-NMR (150 MHz, CDCl_3_) δ = 172.30, 171.37, 161.39, 161.13, 140.65, 140.04, 136.37, 132.34, 130.95, 128.96, 128.93, 128.55, 126.14, 123.58, 120.43, 120.32, 117.70, 114.48, 112.15, 109.86, 64.33, 29.21. MS (MALDI-TOF) [m/z]: Calcd for C_54_H_39_N_5_O_2_, 789.31; Found, 787.942. Anal. Calcd (%) for C_54_H_39_N_5_O_2_: C, 82.11; H, 4.98; N, 8.87. Found; C, 82.10; H, 5.00; N, 8.90.

The synthesis of 9,9′-(5-((9-(4-(4,6-diphenyl-1,3,5-triazin-2-yl)phenoxy)nonyl)oxy)-1,3-phenylene)bis(9H-carbazole) (mCP-9C-TRZ) is as follows.

The synthetic process of mCP-9C-TRZ was similar with that of mCP-3C-TRZ, with mCP-3C-Br replaced with mCP-9C-Br. The clear mucus was obtained with a high yield of 72% (0.58 g). Furthermore, ^1^H-NMR (600 MHz, CDCl_3_) δ = 8.77 (d, *J* = 6.9 Hz, 4H), 8.72 (d, *J* = 11.4 Hz, 2H), 8.15 (d, *J* = 7.7 Hz, 4H), 7.65–7.52 (m, 10H), 7.45 (t, *J* = 7.7 Hz, 4H), 7.40 (t, *J* = 1.7 Hz, 1H), 7.31 (t, *J* = 7.4 Hz, 4H), 7.23 (d, *J* = 1.7 Hz, 2H), 7.04 (d, *J* = 8.8 Hz, 2H), 4.07 (dd, *J* = 13.9, 6.7 Hz, 4H), 1.93–1.80 (m, 4H), 1.56–1.47 (m, 4H), 1.42 (m, 4H), 1.34 (m, 1H), 1.28 (m, 1H). Furthermore, ^13^C-NMR (150 MHz, CDCl_3_) δ = 171.40, 171.25, 163.17, 161.57, 140.57, 139.97, 136.15, 132.02, 131.03, 128.98, 128.59, 128.53, 126.13, 123.54, 120.42, 120.31, 117.32, 114.52, 112.12, 109.88, 68.75, 68.15, 31.45, 30.21, 29.47, 29.33, 29.20, 29.17, 26.04. MS (MALDI-TOF) [*m/z*]: Calcd for C_60_H_51_N_5_O_2_, 873.40; Found, 871.792. Anal. Calcd (%) for C_60_H_51_N_5_O_2_: C, 82.45; H, 5.88; N, 8.01. Found; C, 82.40; H, 5.90; N, 8.05.

### 2.3. OLED Device Fabrication

OLED devices were fabricated using a clean glass substrate coated with an ITO layer as the anode, with a sheet resistance of 15 Ω cm^−2^ and an active pattern size of 2 × 2 mm^2^. Before device fabrication, the glass substrates were sequentially cleaned in an ultrasonic bath with deionized water, acetone and ethanol three times, and then the ITO substrate was treated with UV-ozone for 30 minutes. The OLED configuration was as follows: ITO/PEDOT:PSS (40 nm)/EML(40 nm)/TPBi (40 nm)/Cs_2_CO_3_ (2 nm)/Al (100 nm), where PEDOT:PSS and Cs_2_CO_3_ acted as the hole and electron injection layers, respectively. The TPBi and Al functioned as the electron transport layers and the cathode, respectively. The PEDOT:PSS was spin-coated directly onto an ITO plate and annealed at 150 °C for 10 min. The EML was dissolved in 1,2-dichloroethane (10 mg mL^−1^), then spin-coated and annealed at 80 °C for 10 min under nitrogen atmosphere. Then, the substrates were moved into a vacuum chamber to deposit TPBi, Cs_2_CO_3_ and Al sequentially using a thermal evaporator. The current density–voltage–luminance characteristics, current efficiency and power efficiency were tested using a Keithley 2636A Sourcemeter coupled with Si photodiodes calibrated with PR655. The EL spectra were collected with a Photo-Research PR655 SpectraScan. All the characterizations were performed at room temperature in ambient conditions without encapsulation. External quantum efficiencies of the devices were calculated assuming a Lambertian emission distribution.

## 3. Results and Discussions

### 3.1. Synthesis and Characterizations

Detailed routes of mCP-3C-TRZ and mCP-9C-TRZ are demonstrated in the Supporting Information, which is analogous to that of mCP-6C-TRZ [31]. As shown in Scheme S1, these three target materials were synthesized with high yields via the simple catalyst-free reaction between the mCP and TRZ derivative moieties. The product of pure white powder was obtained by column chromatography and the chemical structures of mCP-3C-TRZ and mCP-9C-TRZ were fully confirmed by ^1^H NMR, ^13^C NMR, mass spectrometry and elemental analysis see Supplementary Materials (Figures S1–S8). Additionally, it was further purified by recrystallization before being used for device fabrication. As expected, these materials show excellent solubility in various common solvents at room temperature, e.g., 1,2-dichloroethane, dichloromethane, trichloromethane and tetrahydrofuran, manifesting the unique superiority of alkyl chain and empowering them as suitable hosts for constructing solution processable OLEDs. Moreover, the smooth surface morphologies of mCP-3C-TRZ, mCP-6C-TRZ and mCP-9C-TRZ were confirmed by atomic force microscopy (AFM) measurement. As shown in Figure S9 (see Supplementary Materials), the values of root-mean-square (RMS) roughness are 0.426 nm, 0.407 nm and 0.355 nm for mCP-3C-TRZ, mCP-6C-TRZ and mCP-9C-TRZ, respectively, guaranteeing the superior morphological stability for the solution method. The longer the flexible chain, the better the film-forming performance.

### 3.2. Theoretical Calculations

**Figure 2:**
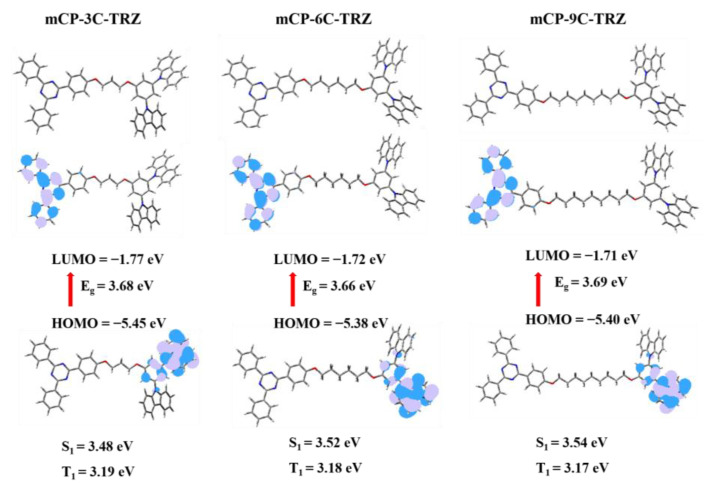
HOMO and LUMO distributions and 3D structure of the optimized ground state calculated by B3LYP/6-31G(d) level for mCP-3C-TRZ, mCP-6C-TRZ and mCP-9C-TRZ.

Theoretical calculations were then employed on mCP-3C-TRZ, mCP-6C-TRZ and mCP-9C-TRZ to simulate molecular structure and electronic transition properties. The optimized ground state (S_0_) was firstly operated by using density functional theory (DFT) with B3LYP/6–31G (d) basis set, which is shown in Figure 2. As one might expect, the distribution of the highest occupied molecular orbital (HOMO) and lowest unoccupied molecular orbital (LUMO) of mCP-3C-TRZ, mCP-6C-TRZ and mCP-9C-TRZ are accordant, where the HOMOs of the above materials are thoroughly distributed on the benzene ring and the adjacent carbazole group and the LUMOs are only located on the triazine unit and two outboard benzene rings. The spatial distribution, according to frontier molecular orbital (FMO) principles, implies that the alkyl chain can only serve as a bridge to break the molecular conjugation and provide a possibility to proceed with intermolecular charge transfer (CT). The calculated optical energy gaps are similar, which are in the range of 3.66–3.69 eV for the three molecules. The adiabatic excited states of the singlet (S_1_) and triplet (T_1_) were simulated based on the optimized ground state by using the time-dependent DFT (TD-DFT) calculations with the same basis set, revealing accordant S_1_ energy of 3.48–3.54 eV and T_1_ energy of 3.17–3.19 eV. That indicates an acceptable enough capacity for these three molecules to be used as the host material. Since the conjugation of the molecular skeleton was completely broken, the oscillator strength was estimated to be 0 eV for all three molecules, indicating the low probability of intramolecular transitions. In addition, the distances between the cores of the mCP and TRZ units are theoretically estimated at 20 Å, 24 Å and 28 Å for mCP-3C-TRZ, mCP-6C-TRZ and mCP-9C-TRZ, respectively, which hampers the possibility of intramolecular spatial CT transition. Therefore, in view of the restricted intramolecular CT transition, mCP-3C-TRZ, mCP-6C-TRZ and mCP-9C-TRZ show local excited states of mCP and TRZ groups in a single-molecule state or monodisperse state.

### 3.3. Photophysical Properties

**Figure 3:**
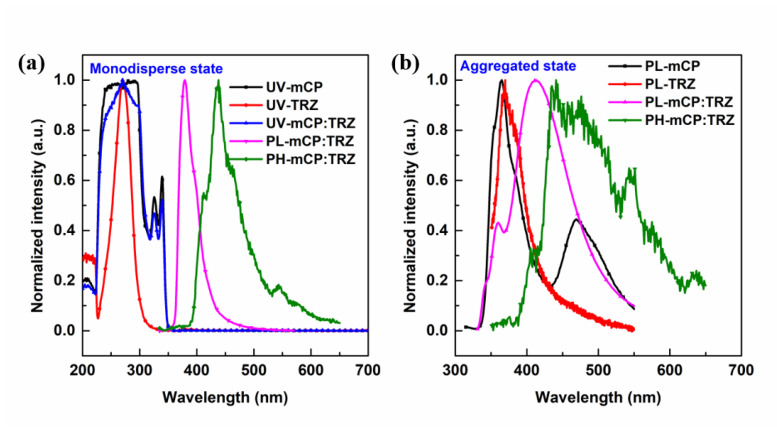
(**a**) Normalized absorption spectra, fluorescence spectra and low temperature phosphorescence spectra (after delay of 100 μs) of mCP, TRZ and mCP:TRZ in toluene (10^−5^ M). (**b**) The normalized fluorescence spectra and low-temperature phosphorescence spectra (after delay of 100 μs) of mCP, TRZ and mCP:TRZ in neat solid films.

Photophysical characterizations of mCP-3C-TRZ, mCP-6C-TRZ and mCP-9C-TRZ, including absorption (UV), fluorescence (PL) and phosphorescence (PH), were, respectively, carried out in a solution and a solid-state film, which, respectively, represented a monodispersed state and aggregated state. Primarily, to obtain a deep understanding of the predominant transition channel of mCP-3C-TRZ, mCP-6C-TRZ and mCP-9C-TRZ, the photophysical properties of mCP and TRZ were characterized. As shown in Figure 3a, the PL emission from the mixture of mCP and TRZ shows a narrow peak in toluene (10^−5^ mol/L), exhibiting strong local excited state (LE) emission. In contrast, the PL emission of the same mixture features two peaks in solid-state film, the former (360 nm) attributed to the emission of a single component (mCP or TRZ), the latter (410 nm) induced by the obvious intermolecular CT transition. It has been reported in the literature that mCP and TRZ molecules could form intermolecular CT-type exciplex [30,31]. In addition, in the aggregated state, mCP could form excimer with adjacent mCP molecules, which could also be observed from Figure 3b.

**Figure 4:**
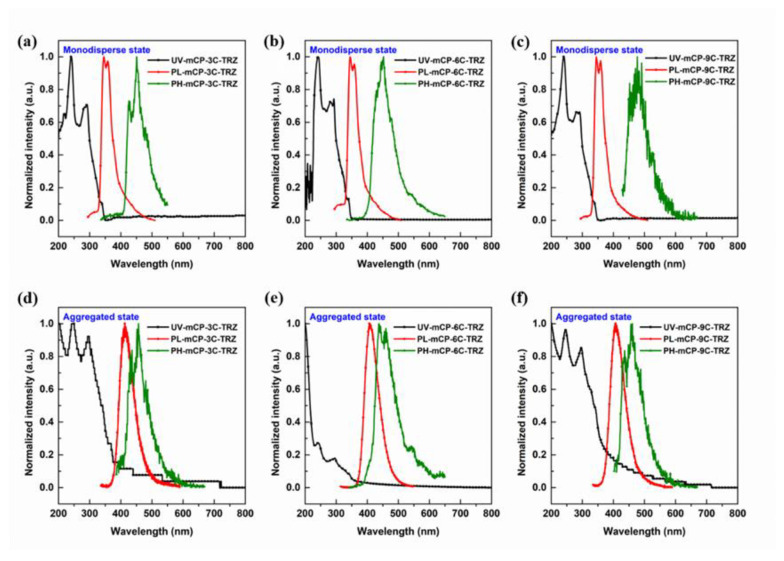
Normalized absorption spectra, fluorescence spectra and low-temperature phosphorescence spectra (after delay 100 μs) of mCP-3C-TRZ, mCP-6C-TRZ and mCP-9C-TRZ in toluene (**a**–**c**) and in solid-state film (**d**–**f**).

Figure 4 displays the PL properties of mCP-3C-TRZ, mCP-6C-TRZ and mCP-9C-TRZ in toluene and neat film. Clearly, the similar absorption spectra of these compounds in the range of 230–350 nm are observed in toluene solution, and no obvious broad absorption from intramolecular CT is visible. Accordant LE-type PL emission is shown for three molecules in toluene, which is consistent with the theoretical calculation that the intramolecular CT transition is restricted due to the significant distance and non-conjugated link between mCP and TRZ. Normally, if there is no new emission band observed from the PL spectra of neat films, it can be concluded that no new species appeared in the singlet state. As shown in Figure 4d, however, different from the phenomenon in toluene in Figure 4a, a distinctly red-shifted and unique fluorescence emission peak appearing at 413 nm is observed for the neat film of mCP-3C-TRZ, which matches well with the PL spectra of mCP:TRZ in Figure 3b. The same phenomenon occurs in the PL spectra of neat films for mCP-6C-TRZ and mCP-9C-TRZ, as displayed in Figure 4e,f, confirming the formation of intermolecular CT. In addition, the PL emission peaks of the neat films are the same for all three molecules, meaning the length of the flexible chain has no impact on intermolecular CT process in an aggregated state. The PH spectra in a monodisperse state and an aggregated state show characteristic vibrational structures, indicating that the triplet of mCP-3C-TRZ, mCP-6C-TRZ and mCP-9C-TRZ comes from the LE state. The S_1_/T_1_ energies of mCP-3C-TRZ, mCP-6C-TRZ and mCP-9C-TRZ obtained from the onset of PL and PH spectra in an aggregated state are 3.28/3.02 eV, 3.29/3.05 eV and 3.30/3.00 eV. Corresponding data were summarized in Table 1. The high triplet energy ensures the ability of being used as host materials during solution-processed OLEDs.

**Figure 5:**
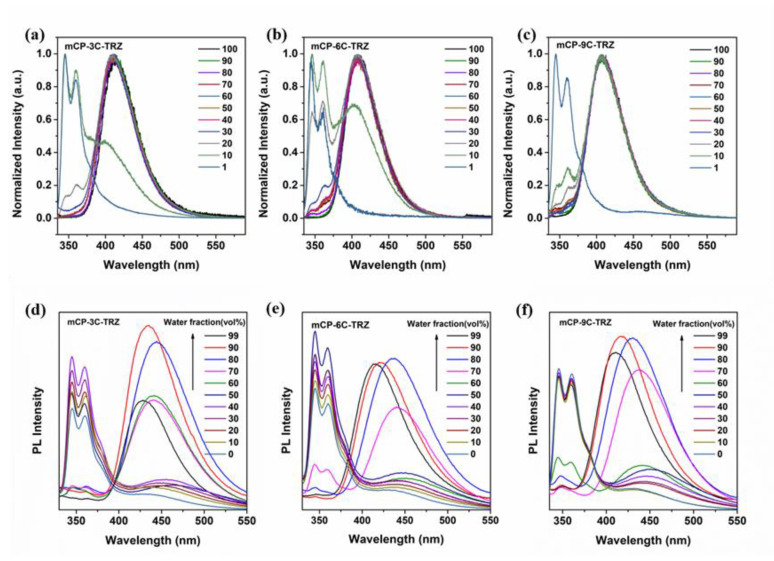
The normalized PL spectra of doped films when mCP-3C-TRZ (**a**), mCP-6C-TRZ (**b**) and mCP-9C-TRZ (**c**) were diluted into PMMA matrix. Additionally, the fluorescence spectra of mCP-3C-TRZ (**d**), mCP-6C-TRZ (**e**) and mCP-9C-TRZ (**f**) in mixed THF/water solutions of 1 × 10^₋5^ M concentration with various water fractions under ambient temperature.

To deepen the understanding of the charge transfer process in an aggregated state, the PL spectra of mCP-3C-TRZ, mCP-6C-TRZ and mCP-9C-TRZ doped in poly(methyl methacrylate) (PMMA) with different concentrations were further investigated. Figure 5a–c demonstrate that, at a low dopant concentration of 1 wt%, three PL spectra show similar LE-type emission from mCP or TRZ. When increasing dopant concentration to 10 wt%, the emission peak at 410 nm gradually appears and this intensity is gradually strengthened from mCP-3C-TRZ to mCP-9C-TRZ. In particular, for mCP-9C-TRZ, the emission of 410 nm is the dominant peak for the PL spectra under the dopant concentration of 10 wt%. To continue to enhance the doped concentration, the LE emission decreases and even vanishes at a high dopant concentration > 70 wt%, and the PL emission of CT process is increased and even becomes the main peak for the neat film of mCP-3C-TRZ, mCP-6C-TRZ and mCP-9C-TRZ. Additionally, the CT-type emission peaks remain nearly consistent in spite of the changed dopant concentration. The above results can be ascribed to the distinctly curtate intermolecular distance, which promotes the intermolecular CT transition in view of the obstruction of intramolecular CT transition. As a consequence, mCP-3C-TRZ, mCP-6C-TRZ and mCP-9C-TRZ with flexible chains to link donor and acceptor units only feature the obvious intermolecular charge transfer process, which provides new insights to analyze aggregate-induced intermolecular interaction.

In addition to the solid-state film, the PL behavior of nanoparticles could reflect the process of intermolecular interaction. Generally, the free motion, including rotation and vibration of each subunit, could lead to the loss of excited state energy in a nonradiative decay manner and quench the fluorescence in solution. Once aggregates form, the nonradiative decay pathways would be intercepted due to such restricted molecular motion. On the other hand, with the formation of aggregates, interactions between molecules are beginning to emerge in view of smaller intermolecular distance. Figure 5d–f display the PL spectra of mCP-3C-TRZ, mCP-6C-TRZ and mCP-9C-TRZ in the mixed solvent of THF/H_2_O with various ratio fractions of deionized water. The PL spectra in pure THF peak at 350 nm and are associated with the emission of LE state, and the emission becomes gradually weaker or even disappears upon the gradual addition of water into the THF solution. Meanwhile, a new broad peak rises from 420 to 450 nm with increasing f_w_, which is identical to the emission of charge transfer state, because it has shown that the intramolecular CT process is absent for mCP-3C-TRZ, mCP-6C-TRZ and mCP-9C-TRZ. This phenomenon that the LE-state emission fades away is because of the gradual utilization of local units to contribute to the occurrence of an intermolecular interaction. Thus, a new emission peak appears at the range of 420–450 nm for mCP-3C-TRZ, mCP-6C-TRZ and mCP-9C-TRZ when intermolecular charge transfer is formed under high enough concentrations of water fractions.

### 3.4. Thermal Stability and Electrochemistry Properties

The measurement of thermogravimetric analysis (TGA) and differential scanning calorimetry (DSC) were conducted under a nitrogen atmosphere to determine the thermal properties of these compounds. As revealed in Figure S10 (see Supplementary Materials), mCP-3C-TRZ and mCP-9C-TRZ show good thermal stability with a decomposition temperature (T_d_, corresponding to 5% weight loss) exceeding 400 °C. Moreover, the glass transition temperatures (T_g_) show the decreasing trend with the length of alkyl chain, 98 °C for mCP-3C-TRZ, 92 °C for mCP-6C-TRZ and 73 °C for mCP-9C-TRZ, respectively. Such poor thermal properties of mCP-9C-TRZ with longer flexible chains could be unsuitable to remain as stable morphological films during the device operation. In contrast, mCP-3C-TRZ with highest T_d_ and T_g_ values would be desirable for good performances of solution-processed OLEDs.

The electrochemical properties of these materials were examined by cyclic voltammetry under the condition of dehydrated dichloromethane as the solvent, Bu_4_NPF_6_ as the supporting electrolyte and the ferrocenium/ferrocene couple (Fc^+^/Fc) as the internal reference (Figure S11 (see Supplementary Materials)). The oxidation peaks with an obvious irreversible behavior are observed for all three compounds, which originate from the electron-donating group. According to the first onset potential of mCP-3C-TRZ, mCP-6C-TRZ and mCP-9C-TRZ, which are 1.04 V, 1.05 V and 1.06 V, respectively; the HOMO energy levels are calculated to be −5.44 eV, −5.45 eV and −5.46 eV. Combined with the optical energy gap, the LUMO energy levels are calculated to be −1.90 eV, −1.91 eV and −1.92 eV for mCP-3C-TRZ, mCP-6C-TRZ and mCP-9C-TRZ, respectively. The similar HOMO and LUMO levels of mCP-3C-TRZ, mCP-6C-TRZ and mCP-9C-TRZ can be ascribed to the identical distribution of their FMOs, as well as the same mCP and TRZ units. Meanwhile, the experimental values are consistent with the calculated values, which indicates a slight influence of the length of the flexible chain in three molecules on their electrochemical properties.

### 3.5. Device Performance

For exploring the influence of the flexible chain on carrier transport and balance, the carrier-only devices of these emitters were fabricated using a structure of ITO|PEDOT:PSS (40 nm)|EML (40 nm)|MoO_3_ (20 nm)|Al (100 nm) for hole-only devices and with a configuration of ITO|Al (50 nm)|EML (40 nm)|TPBi (40 nm)|Cs_2_CO_3_ (2 nm)|Al (100 nm) for electron-only devices, respectively. The current density–voltage (J–V) curves of electron- and hole-only devices are displayed in Figure S12 (see Supplementary Materials). The result shows a greater mobility of the holes than that of the electron for mCP-3C-TRZ, mCP-6C-TRZ and mCP-9C-TRZ, and the hole mobility of these three compounds is similar. When the flexible chain gets shorter, the mobility of the hole and particle gradually becomes synchronized, revealing that mCP-3C-TRZ has excellent bipolar carrier transport and balance properties. Bipolar transporting property suggests these compounds could act as host materials, and balanced carrier mobility implies that mCP-3C-TRZ-based devices have better performance.

**Figure 6:**
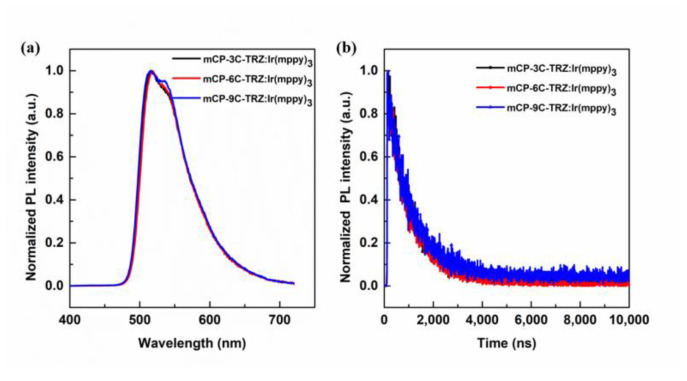
(**a**) The PL spectra and (**b**) transient PL decay spectra of Ir (mppy)_3_ doped into mCP-3C-TRZ, mCP-6C-TRZ and mCP-9C-TRZ films (5 wt%) at room temperature.

To uncover the efficient energy transfer, the tris(2-methylphenylpyridine) iridium (III) (Ir(mppy)_3_) was selected as a phosphorescent guest with a concentration of 5 wt% of mCP-3C-TRZ-, mCP-6C-TRZ- and mCP-9C-TRZ-doped films. As the PL emission profile of host materials is quenched, the PL emission of doped film in Figure 6a only exhibits a characteristic peak of Ir(mppy)_3_ with an excitation wavelength of 380 nm, showing an efficient energy transfer process from the bipolar host to the phosphorescent guest. The photoluminescence quantum yields (PLQYs) are 77%, 75% and 74% for mCP-3C-TRZ-, mCP-6C-TRZ- and mCP-9C-TRZ-doped films. Furthermore, the PL decay curves of the doped films are shown in Figure 6b, presenting two decay components, defined as prompt fluorescence and phosphorescence. The long lifetime is deduced from the T1 state emission from Ir(mppy)_3_. These photophysical measurements make clear that the energy is efficiently transferred from the host to the guest.

**Figure 7:**
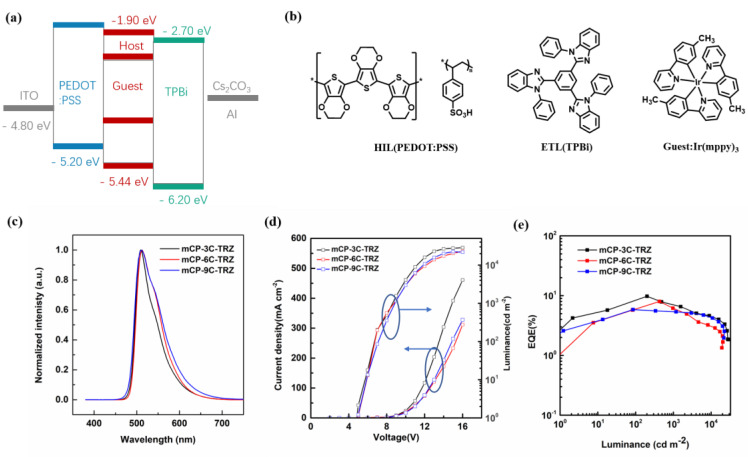
(**a**) Energy diagram of the materials used in the devices, (**b**) chemical structures of organic materials in OLEDs, (**c**) normalized EL spectra in the doped devices, (**d**) current density–voltage–luminance (J–V–L) curves, (**e**) external quantum efficiency versus luminance curves.

Then, three doped OLEDs, with the following configuration: ITO/PEDOT:PSS (40 nm)/emissive layer (host:5 wt% Ir(mppy)_3_ (40 nm)/TPBi (40 nm)/Cs_2_CO_3_ (2 nm)/Al (100 nm), were prepared via solution method, in which ITO was used as the anode material; poly(3,4-ethylenedioxythiophene)-poly(styrene sulfonate) (PEDOT:PSS) was used as the hole-injection layer, 1,3,5-Tris(1-phenyl-1H-benzimidazol-2-yl)benzene (TPBi) and Cs_2_CO_3_ served as the electron transport and electron injection layers, respectively. Finally, aluminum (Al) acted as the cathode. The device configuration and chemical structures of used materials in devices are shown in Figure 7a,b. These doped devices were fabricated under the same conditions. As shown in Figure 7c, the devices exhibited the same EL peak at 512 nm, confirming the efficient energy transfer from host to phosphorescent guest, driven by electric field. Figure 7d shows the turn-on voltages of these devices are in the range of 4.5–5.0 V. As expected, the mCP-3C-TRZ-based device achieved the best performances with an EQE of 9.8 %, which is much better than mCP-6C-TRZ with an EQE of 7.0% and mCP-9C-TRZ with an EQE of 5.8%, as shown in Figure 7e. The corresponding data were summarized in Table 2. Given the similar PLQY, the difference of the device performance could be attributed to that of the mCP-3C-TRZ as the bipolar host material increases hole mobility and balances carriers during the solution process, as well as the stable thermal characteristics.

## 4. Conclusions

In summary, three compounds with a donor–spacer–acceptor structure, were designed and characterized via adjusting the length of the alkyl chain from propane to nonane. The introduction of the alkyl chain not only blocks the molecular conjugation, but also suppresses the intramolecular CT process and generates aggregation-induced intermolecular charge transfer emission. Although the alkyl chain could improve the solubility and film-forming property of materials, the longer alkyl chain lowers the glass transition temperature and induces poor thermal stability. The excited state energy levels are independent of the length of the alkyl chain and all three molecules possess high E_T_, which is beneficial when being used as a host material. Additionally, these three compounds show excellent bipolar carrier transport characteristics and the shorter the alkyl chain, the more balanced the carrier transport. Therefore, when employing mCP-3C-TRZ as a host for the phosphorescent OLEDs, the solution-processed devices realize a maximum EQE_max_ of 9.8%, which is superior to that of mCP-6C-TRZ and mCP-9C-TRZ. This work illustrates molecular engineering tactics to explore novel host materials via utilizing intermolecular aggregation emission for efficient solution-processed OLEDs.

## Figures and Tables

**Table 1:** Basic photophysical and electrochemical parameters of mCP-3C-TRZ, mCP-6C-TRZ, and mCP-9C-TRZ.

Compound	T_g_^a^ (°C)	λ_abs_ † (nm)	λ_em_^b^ (nm)	λ_em_^c^ (nm)	E_g_^d^ (eV)	S_1_^e^/T_1_^f^ (eV)	ΔE_ST_^g^ (eV)	HOMO ^h^ (eV)	LUMO ^i^ (eV)
mCP-3C-TRZ	98	240, 292, 339	345, 360	413	3.65	3.28/ 3.02	0.26	−5.44	−1.90
mCP-6C-TRZ	92	240, 293 339	345, 359	413	3.65	3.29/ 3.05	0.24	−5.45	−1.91
mCP-9C-TRZ	73	240, 291, 339	344, 359	413	3.65	3.30/ 3.00	0.30	−5.46	−1.92

^a^ T_g_: glass transition temperature. † Measured in toluene solution (10^−5^ M) at 300 K. ^b^ Measured in toluene solution (10^−5^ M) at 300 K. ^c^ Measured in the neat films. ^d^ The calculated optical energy gap (E_g_) from the onset of absorption in toluene (10^−5^ M) at 300 K. ^e^ The calculated energy of S_1_ from the onset of PL spectra measured from solid-state film at 300 K. ^f^ The calculated energy of T_1_ from the onset of Phos spectra measured from solid-state film after delayed at 77 K. ^g^ The S_1_-T_1_ energy gap (ΔE_ST_) measured from solid-state film. ^h^ HOMO/^i^ LUMO energy levels estimated by cyclic voltammetry measurement. ***E***_LUMO_ = ***E***_HOMO_ + ***E***_g_. ***E***_HOMO_ (ferrocene) = 4.8 eV [41].

**Table 2:** Device performances of the solution-processed phosphorescent OLEDs.

Emitting Layer	EL ^a^ (nm)	V_on_^b^ (V)	L_max_^c^ (cd m^−2^)	CE_max_^d^ (cd A^−1^)	PE_max_^e^ (lm W^−1^)	EQE_max_^f^ (%)	CIE ^g^ (x, y)
mCP-3C-TRZ: Ir(mppy)_3_	512	4.5	28,589	29.2	13.1	9.8	(0.29, 0.62)
mCP-6C-TRZ: Ir(mppy)_3_	512	4.8	20,190	20.9	9.4	7.0	(0.29, 0.62)
mCP-9C-TRZ: Ir(mppy)_3_	512	4.9	22,600	17.3	7.8	5.8	(0.29, 0.62)

^a^ The stable EL emission spectrum at 10 V. ^b^ Turn-on voltage at the luminescence of 1 cd m^−2^. ^c^ Maximum luminance. ^d^ Maximum current efficiency. ^e^ Maximum power efficiency. ^f^ The maximum external quantum efficiency. ^g^ The Commission International de L’Eclairage (CIE) coordinates at 10 V.

## Data Availability

Data is contained within the article or supplementary material.

## Supplementary Materials

The following supporting information can be downloaded at: https://www.mdpi.com/article/10.3390/molecules27228099/s1, Figure S1: ^1^H-NMR spectrum of mCP-3C-Br; Figure S2: ^1^H-NMR spectrum of mCP-9C-Br; Figure S3: ^1^H-NMR spectrum of mCP-3C-TRZ; Figure S4: ^13^H-NMR spectrum of mCP-3C-TRZ; Figure S5: ^1^H-NMR spectrum of mCP-9C-TRZ; Figure S6: ^13^C-NMR spectrum of mCP-9C-TRZ; Figure S7: Mass spectrum of mCP-3C-TRZ; Figure S8: Mass spectrum of mCP-9C-TRZ; Figure S9: AFM topographic images of the solution-processed neat films of mCP-3C-TRZ, mCP-6C-TRZ and mCP-9C-TRZ; Figure S10: (a) TGA curves and DSC curves of mCP-3C-TRZ (a,c) and mCP-6C-TRZ (b,d) at a heating rate of 10 °C min^−1^ under nitrogen; Figure S11: The cyclic voltammetry of mCP-3C-TRZ, mCP-6C-TRZ and mCP-9C-TRZ at room temperature; Figure S12: IV characteristics (a) of electron-only devices with a configuration of ITO|Al (50 nm)|EML (40 nm)|TPBi (40 nm) |Cs_2_CO_3_(2 nm) |Al (100 nm), and IV characteristics (b) of hole-only devices with configurations of ITO|PEDOT:PSS (40 nm)|EML (40 nm)|MoO_3_ (20 nm)|Al (100 nm) based on mCP-3C-TRZ, mCP-6C-TRZ and mCP-9C-TRZ; Experimental Section: The synthesis procedure.
