# Supplementary material for: Aggregation-Induced Intermolecular Charge Transfer Emission for Solution-Processable Bipolar Host Material via Adjusting the Length of Alkyl Chain
Source: Molecules. 2022 Nov 21;27(22):8099. doi: 10.3390/molecules27228099 (PMC9698787; doi:10.3390/molecules27228099)
Supplement: Supplementary file 1 [file molecules-27-08099-s001.zip › molecules-2023395-supplementary.pdf]

Supporting information

Aggregation-Induced Intermolecular Charge Transfer Emission for Solution-Processable Bipolar Host Material via Adjusting the Length of Alkyl Chain

Wei Jiang *, Guimin Zhao, Wenwen Tian and Yueming Sun

School of Chemistry and Chemical Engineering, Southeast University, Nanjing 211189, China

* Correspondence: jiangw@seu.edu.cn

1. Experimental Section

The synthesis procedure

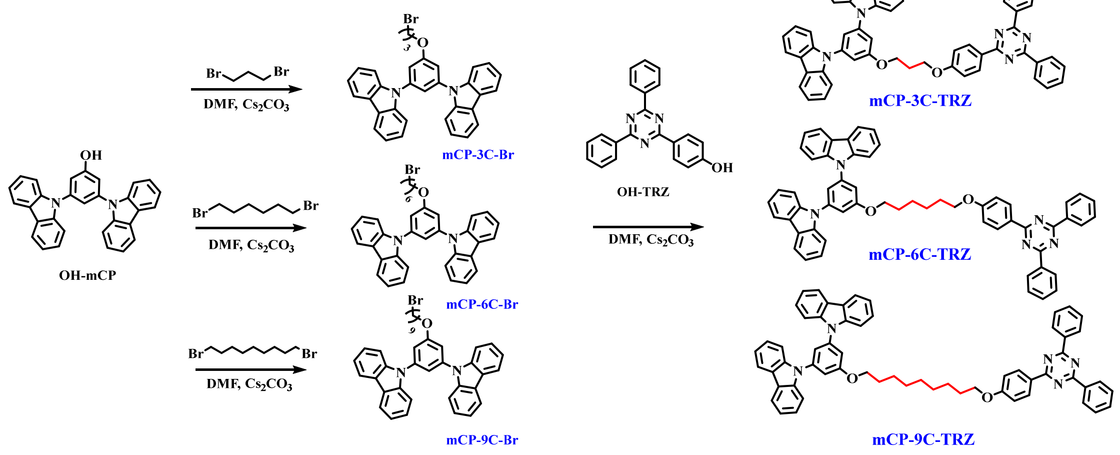

Scheme S1. Synthesis routes of mCP-3C-TRZ, mCP-6C-TRZ and mCP-9C-TRZ.

All materials and solvents were obtained from commercial suppliers and used without further purification. The 3,5-di(9H-carbazol-9-yl)phenol (OH-mCP), 4-(4,6-diphenyl-1,3,5-triazin-2-yl)phenol (OH-TRZ) and 9,9'-(5-((6-(4-(4,6-diphenyl-1,3,5-triazin-2-yl)phenoxy)hexyl)oxy)-1,3-phenylene)bis(9H-carbazole) (mCP-6C-TRZ) were obtained according to the literature [1-3].

2. Supplementary Figures

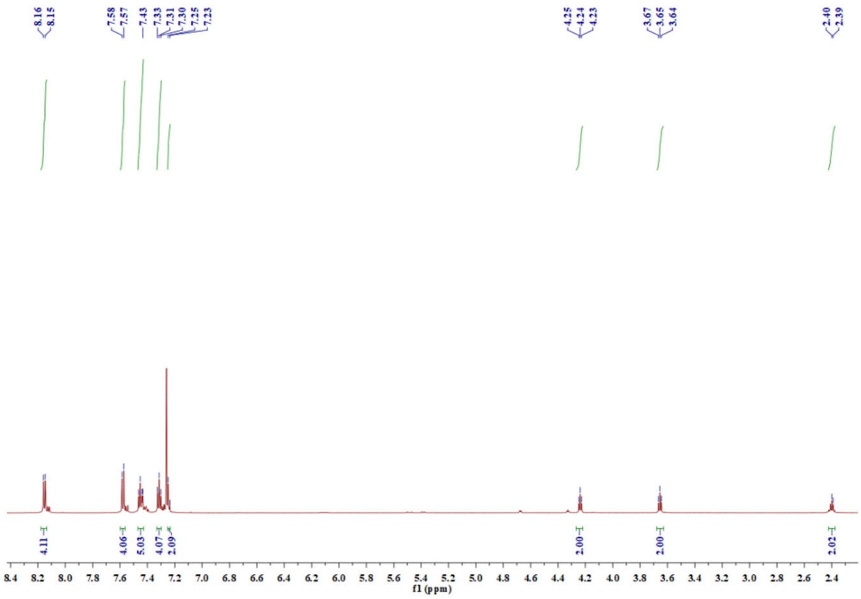

Figure S1. ^{1}H-NMR spectrum of mCP-3C-Br.

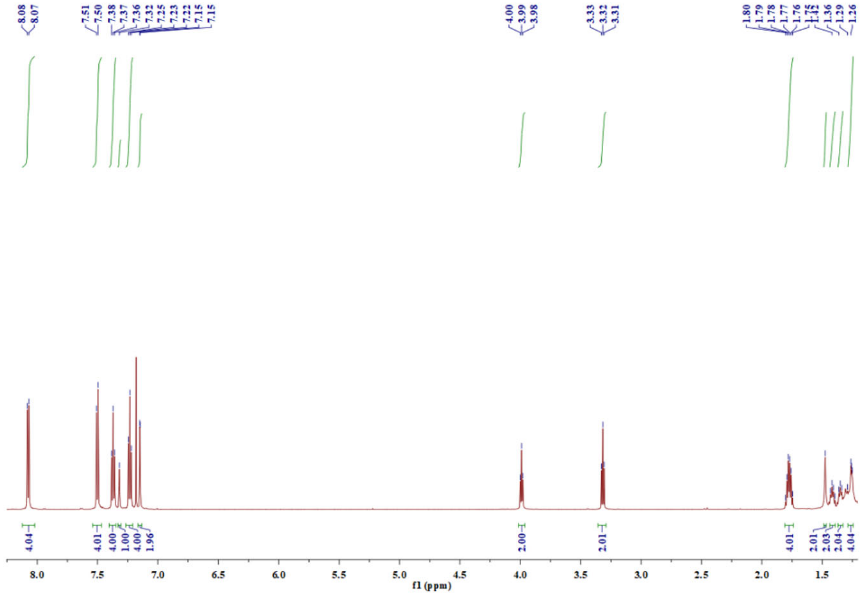

Figure S2. ^{1}H-NMR spectrum of mCP-9C-Br.

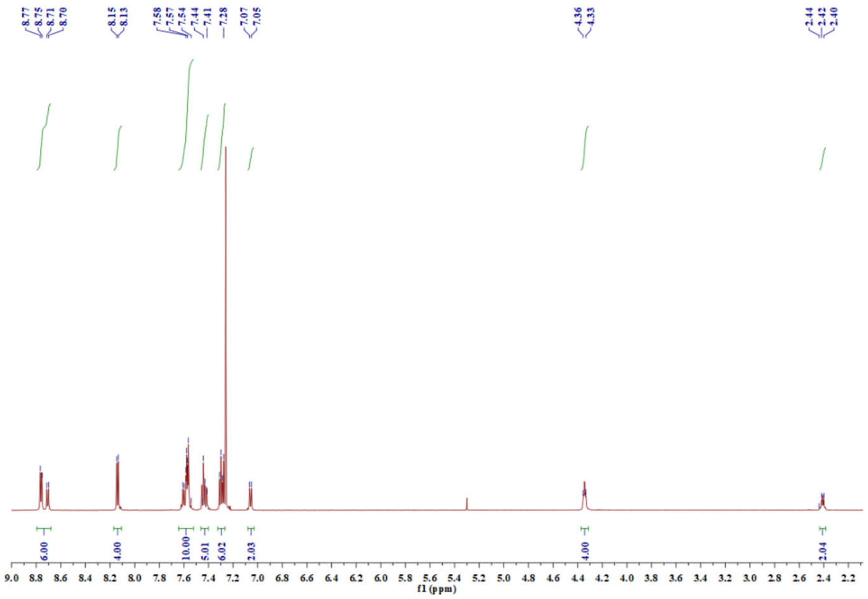

Figure S3. ^{1}H-NMR spectrum of mCP-3C-TRZ.

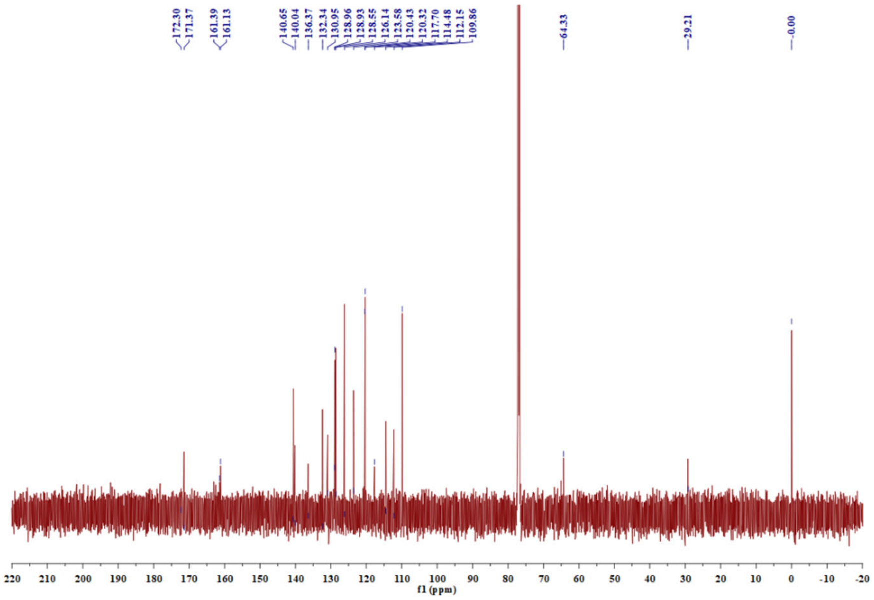

Figure S4. [13]H-NMR spectrum of mCP-3C-TRZ.

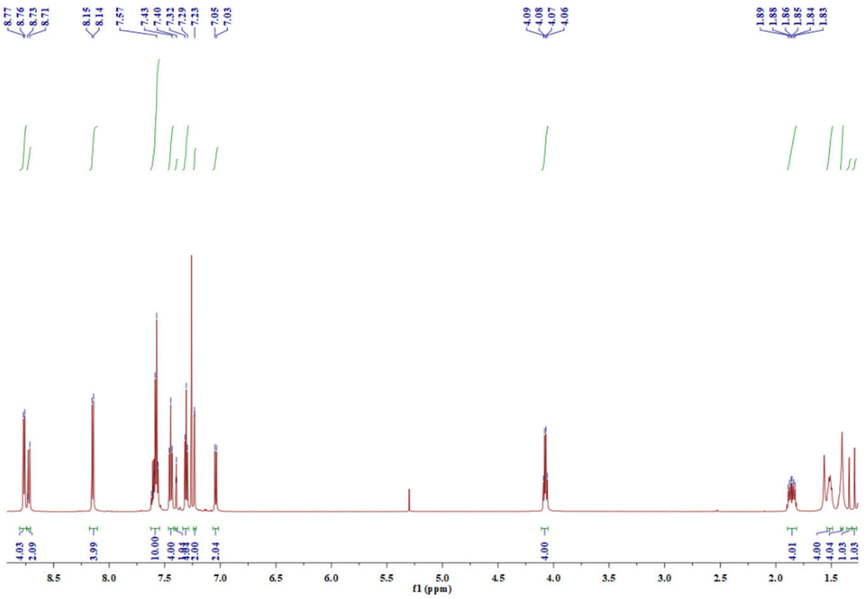

Figure S5. ^{1}H-NMR spectrum of mCP-9C-TRZ.

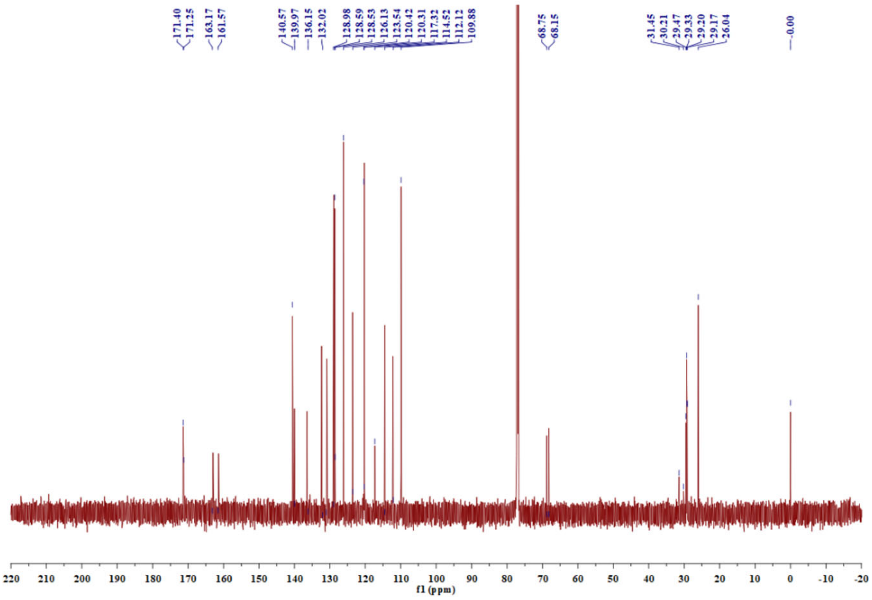

Figure S6. ^{13}C-NMR spectrum of mCP-9C-TRZ.

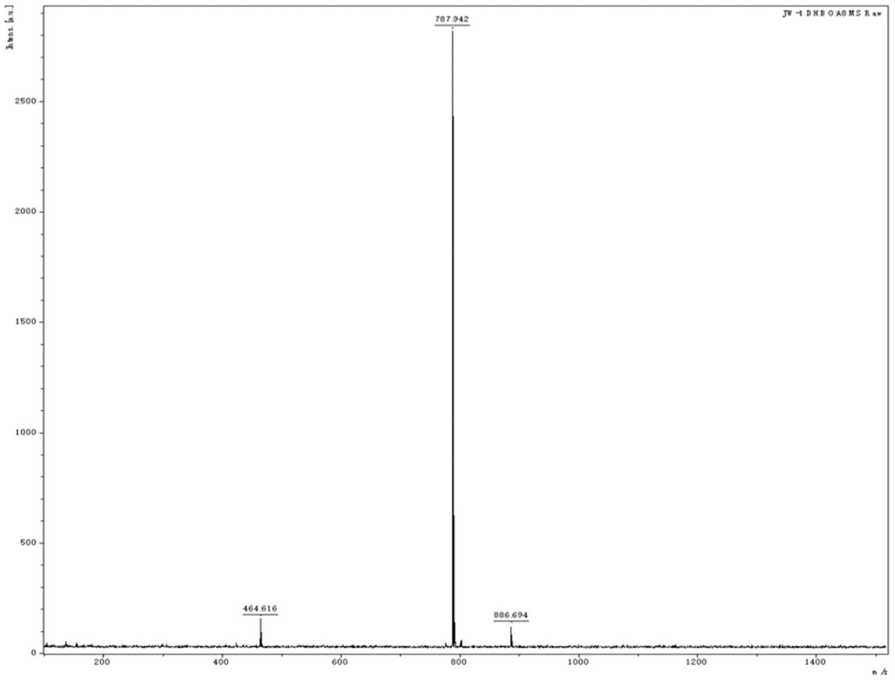

Figure S7. Mass spectrum of mCP-3C-TRZ.

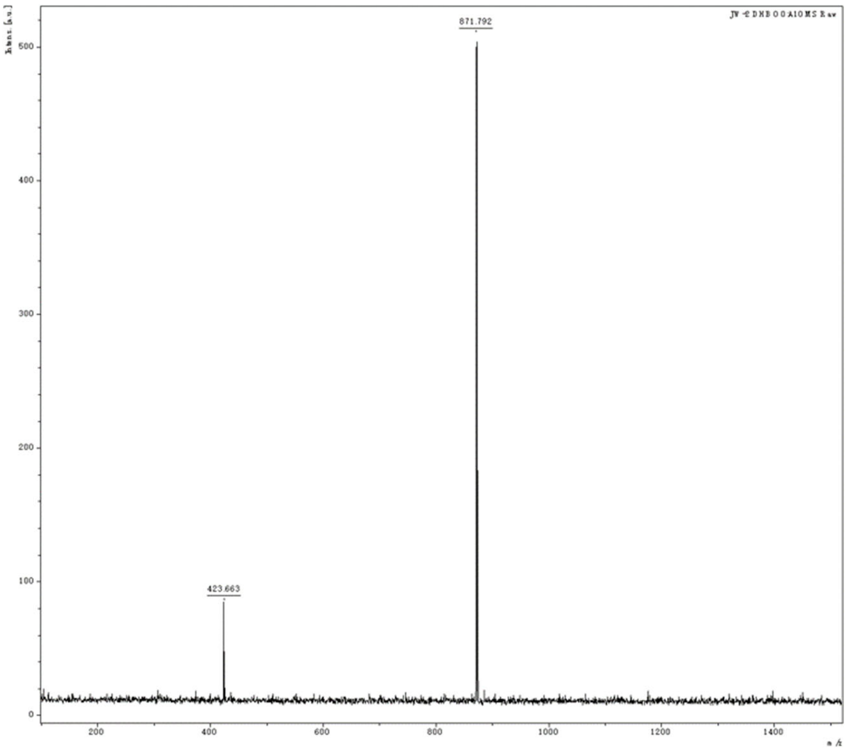

Figure S8. Mass spectrum of mCP-9C-TRZ.

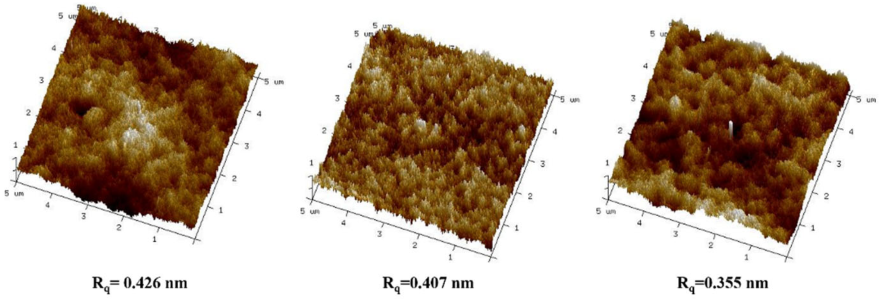

Figure S9. AFM topographic images of the solution-processed neat films of mCP-3C-TRZ, mCP-6C-TRZ and mCP-9C-TRZ.

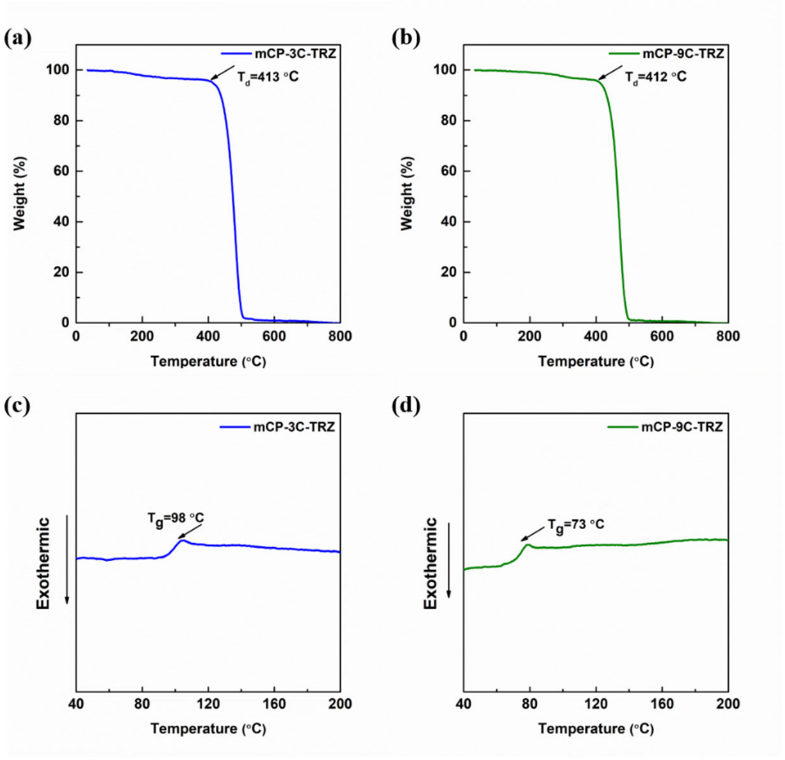

Figure S10. (a) TGA curves and DSC curves of mCP-3C-TRZ (a, c) and mCP-6C-TRZ (b, d) at a heating rate of 10 °C min^{-1} under nitrogen.

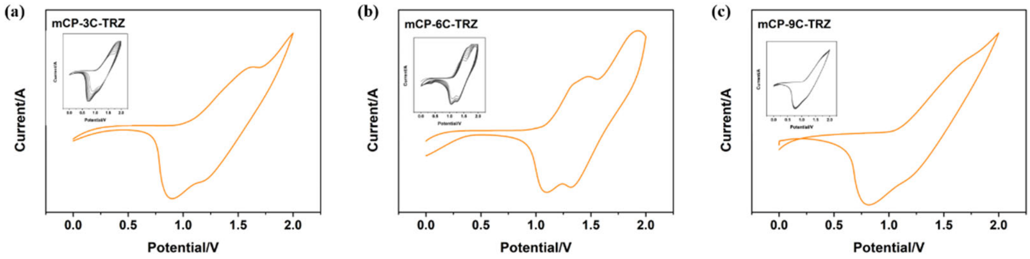

Figure S11. The cyclic voltammetry of mCP-3C-TRZ, mCP-6C-TRZ and mCP-9C-TRZ at room temperature.

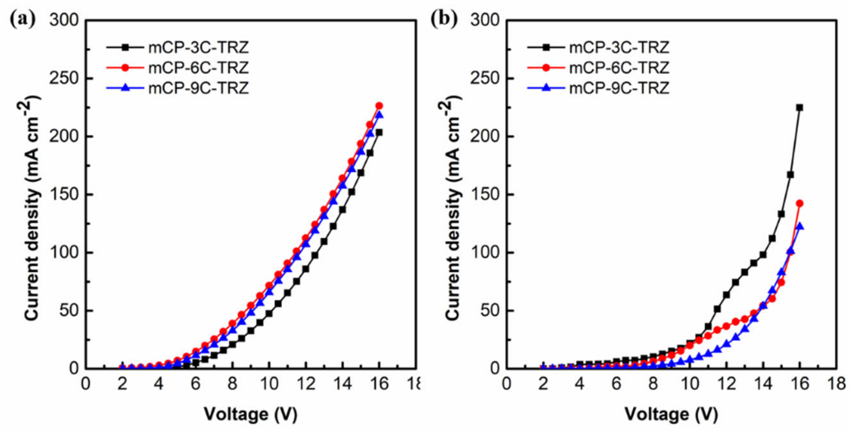

Figure S12. IV characteristics (a) of electron-only devices with a configuration of ITO|Al (50 nm)|EML (40 nm)|TPBi (40 nm) |Cs_2CO_3(2 nm) |Al (100 nm), and IV characteristics (b) of hole-only devices with configurations of ITO|PEDOT:PSS (40 nm)|EML (40 nm)|MoO_3 (20 nm)|Al (100 nm) based on mCP-3C-TRZ, mCP-6C-TRZ and mCP-9C-TRZ.

3. References

1. Sun, K.; Sun, Y.; Tian, W.; Liu, D.; Feng, Y.; Sun, Y.; Jiang, W. Thermally activated delayed fluorescence dendrimers with exciplex-forming dendrons for low-voltage-driving and power-efficient solution processed OLEDs. *J. Mater. Chem. C* **2018**, *6*, 43-49.
2. Zhao, G.; Liu, D.; Tian, W.; Jiang, W.; Sun, Y. Rational molecular design of novel host material combing intra- and intermolecular charge transfers for efficient solution-processed organic light-emitting diodes. *Dyes Pigments* **2020**, *175*, 108188.
3. Zhao, G.; Wang, B.; Liu, D.; Ma, D.; Chen, H.; Tian, W.; Ban, X.; Jiang, W.; Sun, Y. Aggregation induced intermolecular charge transfer in simple nonconjugated donor-acceptor system. *Org. Electron*. **2021**, *99*, 106309.
